# Neurological Benefits of Seaweed-Derived Compounds

**DOI:** 10.3390/md24010031

**Published:** 2026-01-08

**Authors:** Leonel Pereira, Ana Valado

**Affiliations:** 1 Centre for Functional Ecology (CFE), Marine Resources, Conservation and Technology, Marine Algae Lab, Associate Laboratory TERRA, Department of Life Sciences, University of Coimbra, 3000-456 Coimbra, Portugal; 2 Polytechnic University of Coimbra, Rua da Misericórdia, Lagar dos Cortiços, S. Martinho do Bispo, 3045-093 Coimbra, Portugal; valado@estesc.ipc.pt; 3 H&TRC—Health & Technology Research Center, Coimbra Health School, Polytechnic University of Coimbra, Rua 5 de Outubro, 3045-043 Coimbra, Portugal; 4 Research Center for Natural Resources, Environment and Society (CERNAS), Polytechnic University of Coimbra, Bencanta, 3045-601 Coimbra, Portugal; 5 MARE—Marine and Environmental Sciences Centre/ARNET—Aquatic Research Network, University of Coimbra, 3000-456 Coimbra, Portugal

**Keywords:** seaweed, marine bioactive compounds, neuroprotection, neurodegenerative diseases, algal extracts

## Abstract

Seaweed represents a diverse group of marine organisms rich in bioactive compounds that have attracted interest for their potential relevance in neurological research. Recent studies highlight their ability to modulate neuroinflammation, oxidative stress, synaptic plasticity, and pathways implicated in neurodegeneration in preclinical models. Extracts from brown, red, and green algae contain polysaccharides, polyphenols, carotenoids, and fatty acids that exhibit neuroprotective, antioxidant, and anti-inflammatory activities in vitro and in vivo, although these findings remain limited to experimental systems. This review synthesizes current evidence on the neurological activities of seaweed-derived compounds, emphasizing mechanistic findings while clearly distinguishing between experimental observations and unvalidated clinical implications. Challenges related to bioavailability, pharmacokinetics, safety, and clinical translation are discussed, alongside considerations for future research. Evidence in humans remains scarce and indirect, and no seaweed-derived compound has demonstrated neuroprotection or disease-modifying effects in clinical settings.

## 1. Introduction

Marine medicine has emerged as a dynamic field that explores the therapeutic potential of marine organisms, particularly seaweed, which contain structurally diverse bioactive compounds of growing scientific interest. Seaweeds are abundant in coastal ecosystems and possess a diverse array of bioactive compounds, including polysaccharides, polyphenols, peptides, and essential minerals. Although these compounds exhibit pharmacological activity in experimental systems, their relevance for human neurological health remains uncertain and unvalidated [1].

In recent years, there has been a surge of interest in natural neuroprotective agents, driven by the global rise in neurodegenerative diseases such as Alzheimer’s and Parkinson’s. Conventional treatments often fall short in halting disease progression or reversing neuronal damage, prompting researchers to investigate alternative strategies. Seaweed-derived compounds (see Table 1), particularly polyphenols and sulfated polysaccharides, have shown potential in modulating oxidative stress, inflammation, and neuronal signaling pathways in preclinical systems, although these mechanisms have not been demonstrated in humans. However, these effects have not been demonstrated in humans, and their translational relevance remains speculative. Studies have highlighted the antioxidant and anti-inflammatory properties of marine macroalgae, suggesting possible roles in mitigating neurodegenerative processes in controlled laboratory settings. Importantly, findings from cell and animal models cannot be extrapolated to humans and do not constitute evidence of neuroprotection or disease-modifying effects in clinical contexts [2].

**Figure 1:**
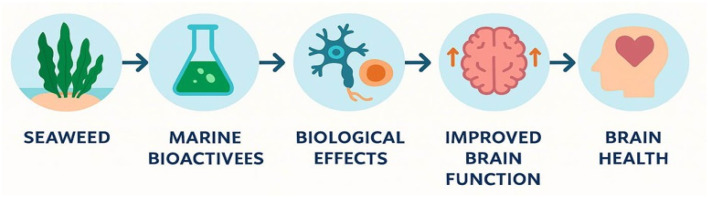
From seaweed to improved brain function. This figure provides a conceptual overview; however, it does not represent quantitative data.

The scope of this review was to synthesize current findings on the neuroprotective effects of seaweed-derived bio-actives and to explore their mechanisms of action within neurological contexts. Specifically, it aims to (i) identify key compounds with neuroprotective potential, (ii) examine their biochemical interactions and pathways relevant to brain health, and (iii) highlight gaps in existing research to guide future investigations. By consolidating evidence from pharmacological, biochemical, and clinical studies, this review seeks to establish a foundation for the development of marine-based interventions targeting brain function (Figure 1) and neurodegeneration [1,11,12].

## 2. Methodology

This review was conducted using structured searches of PubMed, Scopus, Web of Science, and Science Direct, acknowledging that the search strategy was not fully systematic and may not capture all relevant studies. Search terms included ‘seaweed neuroprotection’, ‘fucoidan microglia’, ‘phlorotannin Alzheimer’, ‘fucoxanthin neuroinflammation’, ‘marine polyphenols CNS’, and related combinations, although terminology varies considerably across studies, which may limit retrieval completeness. The time frame covered studies published between 2000 and 2025, but relevant earlier foundational studies were also considered when necessary for mechanistic context.

Inclusion criteria were: (i) peer-reviewed articles, (ii) studies involving marine macroalgae or their isolated compounds, and (iii) relevance to neurological mechanisms or neurodegenerative disease models, recognizing that study heterogeneity limits direct comparison. Exclusion criteria included non-marine sources, studies without neurological endpoints, and non-peer-reviewed material; however, inconsistent reporting across studies may have led to inadvertent omission of relevant data.

Articles were screened by title, abstract, and full text to ensure scientific relevance, although no formal risk-of-bias assessment was performed, which limits the ability to evaluate study quality systematically.

## 3. Seaweed Diversity and Bioactive Constituents

Marine macroalgae, commonly referred to as seaweed, are taxonomically grouped into three major classes: brown algae (Phaeophyceae), red algae (Rhodophyta), and green algae (Chlorophyta). These groups differ in pigmentation, habitat, and biochemical composition, which directly influences their biochemical properties and the variability of their reported biological activities.

Brown algae, such as *Fucus vesiculosus* and *Saccharina japonica* (formerly *Laminaria japonica*), are rich in fucoidan, a sulfated polysaccharide that exhibits anti-inflammatory and antioxidant activity in preclinical models. They also contain phlorotannins, a class of polyphenols unique to brown algae, are rich in fucoidan, a sulfated polysaccharide that exhibits anti-inflammatory and antioxidant activity in preclinical models. Another notable compound is fucoxanthin, a carotenoid with anti-apoptotic and anti-inflammatory effects that may protect neurons from oxidative damage [13,14].

**Figure 2:**
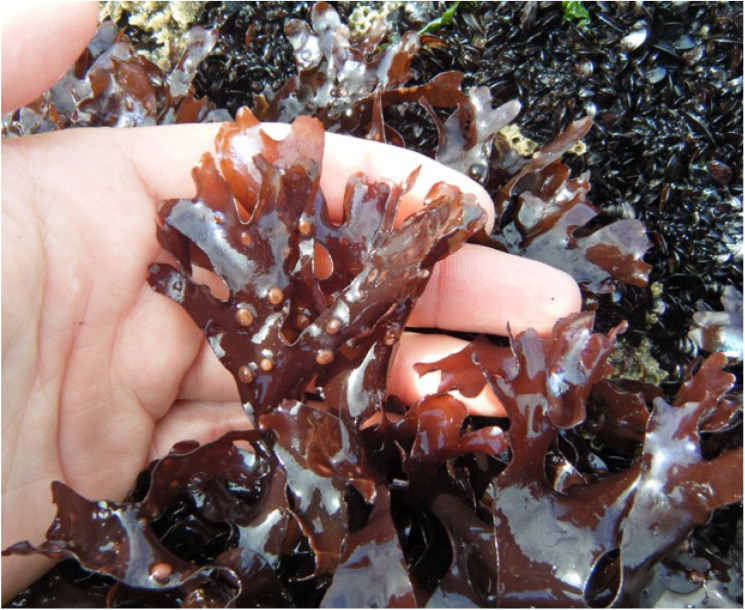
*Chondrus crispus* (Rhodophyta). Note: Figures illustrating representative species of red, brown, and green algae are intentionally presented separately. Each figure appears within the subsection describing its respective algal group, allowing readers to directly associate morphological characteristics with the bioactive compounds discussed in that section. Merging these images into a single figure would disrupt the logical flow and reduce clarity for readers unfamiliar with algal taxonomy.

Red algae, including *Chondrus crispus* (Figure 2) and *Sarcopeltis skottsbergii* (formerly *Gigartina skottsbergii*), are distinguished by their high content of carrageenan, a sulfated galactan with immunomodulatory effects in vitro, although their relevance for neurological outcomes remains uncertain. Red algae also contain bioactive peptides and fatty acids that may influence cellular signaling and membrane properties in experimental systems [9].

Green algae, such as *Ulva lactuca*, are less studied but still valuable. They produce ulvan, a sulfated polysaccharide with antioxidant and anti-inflammatory properties. Green algae also contain essential fatty acids and bioactive peptides that may influence neurotransmission and neurogenesis [15].

Across all three groups, seaweeds are a source of polyunsaturated fatty acids (PUFAs), including Omega-3 and Omega-6, which are crucial for maintaining neuronal membrane fluidity and function. Additionally, bioactive peptides derived from enzymatic hydrolysis of seaweed proteins have shown promise in modulating neuroinflammation and oxidative stress [16]. These compounds collectively contribute to the neuroprotective potential of seaweed, supporting interest in their potential inclusion in functional foods and nutraceuticals, although evidence for neurological benefits in humans is lacking [17].

Although seaweed-derived compounds span diverse chemical classes, the strength of mechanistic evidence varies considerably. Polysaccharides, polyphenols, carotenoids, peptides, sterols, and fatty acids each act through distinct biochemical pathways, yet many findings remain preliminary and dependent on the extraction method, species variability, and experimental model. A critical evaluation of these mechanisms is essential, as many findings remain preliminary and dependent on extraction method, species variability, and experimental models.

Seaweeds are rich in a diverse array of bioactive compounds that contribute to their neuroprotective potential. Among the most studied constituents are polysaccharides, polyphenols, carotenoids, fatty acids, and peptides, each offering distinct biochemical properties relevant to brain health [18]. Polysaccharides such as fucoidan and carrageenan are prominent in brown and red algae, respectively. Fucoidan, found in species like *Fucus vesiculosus* and *Undaria pinnatifida*, exhibits antioxidant, anti-inflammatory, and anti-apoptotic activities that may protect neurons from oxidative damage and neuroinflammation [19].

In addition to the compounds described above, recent studies have identified arachidonic acid from the red alga *Gracilariopsis chorda* as a bioactive molecule with direct effects on neuronal structure and function. This fatty acid has been shown to promote dendritic filopodia formation, enhance spine dynamics, and potentiate synaptic plasticity in hippocampal neurons, suggesting a role in activity-dependent remodeling of neural circuits [20].

Polyphenols, particularly phlorotannins, are unique to brown algae and have garnered attention for their potent antioxidant capacity. These compounds can scavenge reactive oxygen species and inhibit neurotoxic enzymes, contributing to the prevention of neurodegenerative processes [21,22].

Carotenoids like fucoxanthin, also found in brown algae, have demonstrated neuroprotective effects through their ability to modulate oxidative stress and inflammation. Fucoxanthin has been shown to enhance mitochondrial function and reduce neuronal apoptosis in experimental models [7].

Fatty acids, especially polyunsaturated fatty acids (PUFAs) such as Omega-3 and Omega-6, are essential for maintaining neuronal membrane integrity and fluidity. Seaweed provides a plant-based source of these lipids, which are critical for synaptic function and neurogenesis [23].

Peptides derived from enzymatic hydrolysis of seaweed proteins have shown promise in modulating neuroinflammation and oxidative stress. These bioactive peptides may act on neurotransmitter systems and support cognitive function [24].

Together, these compounds form a diverse biochemical profile that warrants further investigation, although current evidence does not establish seaweed as a source of clinically validated neuroprotective agents (Table 2).

The strength of evidence varies considerably among compound classes, and many findings remain preliminary, inconsistent, or dependent on specific experimental conditions. Variability in extraction methods, species composition, seasonal factors, geographic origin, and analytical approaches limit direct comparison across studies and complicates reproducibility.

## 4. Mechanisms of Neurological Activity

Neurological activity is influenced by oxidative stress and inflammation, two interconnected processes frequently examined in experimental models of neurodegenerative disease, although their modulation by seaweed-derived compounds remains largely unvalidated in humans. The brain, with its high metabolic demand and lipid-rich environment, is particularly vulnerable to reactive oxygen species (ROS) generated during mitochondrial respiration, although most evidence regarding the modulation of these processes by seaweed-derived compounds comes from in vitro or animal studies. Antioxidant defense systems, including enzymatic regulators such as superoxide dismutase, catalase, and glutathione peroxidase, act to neutralize ROS and maintain neuronal integrity, although studies assessing the influence of seaweed-derived compounds on these pathways are largely limited to preclinical models. The glutathione system, in particular, represents a critical line of defense, detoxifying peroxides and preserving redox homeostasis, although studies examining how seaweed-derived compounds influence this pathway remain largely restricted to preclinical models. At the transcriptional level, the nuclear factor erythroid 2–related factor 2 (Nrf2) pathway orchestrates the expression of antioxidant response elements, although evidence that seaweed-derived compounds modulate this pathway is largely restricted to in vitro and animal studies.

Fucoidan and ulvan have been reported to modulate microglial activation by inhibiting TLR4-dependent NF-κB signaling in experimental models, although the relevance of these findings for human neuroinflammation remains unconfirmed. This results in reduced secretion of TNF-α, IL-1β, and IL-6 while promoting anti-inflammatory mediators such as IL-10 and TGF-β in experimental models, although these immunomodulatory effects have not been confirmed in human studies. These effects shift microglia from a pro-inflammatory M1 phenotype toward a more anti-inflammatory M2-like profile in experimental models [40], although the extent to which such polarization occurs in humans remains uncertain.

Parallel to oxidative stress, neuroinflammation constitutes a major contributor to neuronal dysfunction, although most evidence regarding its modulation by seaweed-derived compounds derives from in vitro and animal studies. Microglia, the resident immune cells of the central nervous system, are essential for maintaining homeostasis through debris clearance and synaptic remodeling, although most studies evaluating the effects of seaweed-derived compounds on microglial function rely on in vitro or animal models. However, chronic microglial activation leads to sustained release of pro-inflammatory cytokines such as tumor necrosis factor-alpha (TNF-α), interleukin-1 beta (IL-1β), and interleukin-6 (IL-6), which can impair synaptic plasticity and contribute to neuronal loss in experimental models, although the extent to which these processes occur in humans remains uncertain. Anti-inflammatory cytokines, including interleukin-10 (IL-10) and transforming growth factor-beta (TGF-β), counterbalance these effects and support repair processes in experimental models, although their modulation by seaweed-derived compounds has not been demonstrated in humans. The nuclear factor kappa-light-chain-enhancer of activated B cells (NF-κB) pathway serves as a central regulator of inflammatory gene expression, and its inhibition reduces neuroinflammatory cascades in experimental models [41], although evidence for similar effects in humans remains limited.

Fucoidan and ulvan suppress microglial activation by inhibiting TLR4-dependent NF-κB signaling in experimental models, reducing the release of TNF-α, IL-1β, and IL-6 while enhancing anti-inflammatory mediators such as IL-10 and TGF-β. These changes promote a shift from a pro-inflammatory M1 phenotype toward a more anti-inflammatory M2-like profile, although the relevance of this polarization for human neuroinflammation remains uncertain.

Importantly, oxidative stress and inflammation are not isolated phenomena but mutually reinforcing processes. ROS can activate NF-κB, thereby amplifying inflammatory signaling, while inflammatory mediators further increase ROS production, creating a vicious cycle that accelerates neuronal damage in experimental models. This interplay highlights the potential value of interventions that target both pathways, although their efficacy in humans remains to be demonstrated. Natural compounds, particularly marine-derived polyphenols and flavonoids, have attracted attention for their dual antioxidant and anti-inflammatory properties in preclinical studies. Polyphenols modulate Nrf2 activation while suppressing NF-κB signaling in vitro and in vivo, but evidence for synergistic neuroprotection in humans is lacking [42,43]. Similarly, baicalein, a flavonoid with marine analogues, reduces oxidative stress and inflammation by targeting both the Nrf2 and NF-κB pathways in experimental systems [40], although its translational relevance remains uncertain.

The integration of antioxidant and anti-inflammatory pathways is essential for maintaining synaptic integrity, preventing neuronal apoptosis, and supporting cognitive function. From a translational perspective, dietary supplementation with polyphenol-rich marine algae, Omega-3 fatty acids, and other bioactive compounds has been proposed as a potential strategy for neuroprotection, although clinical evidence remains limited. These interventions mitigate oxidative and inflammatory damage in experimental models and may promote resolution of inflammation through specialized pro-resolving mediators derived from marine lipids [44], but their efficacy in humans has yet to be established. As research advances, marine bio-actives may offer novel therapeutic avenues for the management of neurodegenerative diseases, provided that future studies validate their safety, bioavailability, and mechanistic relevance in humans.

The modulation of neurotransmission and synaptic plasticity represents a fundamental mechanism underlying cognitive processes such as learning and memory. Synaptic plasticity refers to the ability of synapses to strengthen or weaken over time in response to activity, ensuring adaptability of neuronal networks. At the molecular level, neurotransmission is regulated by the release of excitatory and inhibitory transmitters, receptor sensitivity, and intracellular signaling cascades. Long-term potentiation (LTP) and long-term depression (LTD) are the most studied forms of synaptic plasticity, both dependent on glutamatergic signaling through NMDA and AMPA receptors. These processes involve calcium influx, activation of kinases such as CaMKII, and downstream transcriptional changes that consolidate synaptic modifications [45], although most studies investigating the modulation of these pathways by marine-derived compounds remain limited to in vitro and animal models.

Beyond classical neurotransmitters, neuromodulators such as dopamine, serotonin, and acetylcholine exert a profound influence on synaptic plasticity. Dopamine enhances LTP in reward-related circuits, while acetylcholine modulates attention and learning by regulating cortical excitability. Emerging evidence also highlights the role of gaseous transmitters such as hydrogen sulfide (H_2_S), which modulates NMDA receptor activity and neurotransmitter release in experimental models, thereby contributing to memory-related processes and synaptic resilience [46], although these effects have not yet been confirmed in humans.

Glial cells, once considered passive support elements, are now recognized as active modulators of synaptic transmission. Astrocytes regulate synaptogenesis, maintain excitatory–inhibitory balance, and release gliotransmitters that influence neuronal excitability. Microglia contribute to synaptic pruning and homeostasis through their surveillance and remodeling functions, while oligodendrocyte precursor cells have recently been implicated in shaping synaptic connectivity and plasticity in experimental models [47]. The extracellular matrix also plays a crucial role by providing structural support and regulating receptor mobility, thereby influencing the stability and adaptability of synapses [48], although most studies examining how marine-derived compounds affect these processes remain limited to in vitro and animal systems.

Plasticity is not limited to homosynaptic changes; heterosynaptic plasticity ensures network-wide balance by adjusting synaptic weights across interconnected neurons. This mechanism prevents runaway excitation and maintains homeostasis, which is critical for stable cognitive function [49]. Together, these processes highlight the intricate interplay between neurotransmitters, neuromodulators, glial cells, and extracellular components in shaping synaptic plasticity, although most studies investigating how marine-derived compounds influence these mechanisms remain restricted to in vitro and animal models.

From a therapeutic perspective, modulation of neurotransmission and synaptic plasticity offers potential avenues for intervention in neurological disorders. Strategies aimed at enhancing beneficial plasticity or dampening maladaptive changes, such as pharmacological modulation of NMDA receptors, targeting glial signaling, or employing marine-derived bio-actives with neuro-modulatory properties have shown promise in experimental models, although evidence for their efficacy in humans remains limited. These approaches may ultimately contribute to neuroprotection and cognitive support, but further clinical validation is required before translational applications can be established.

Neurogenesis and neuronal survival are fundamental processes that sustain brain plasticity and cognitive function throughout life. Neurogenesis, the generation of new neurons from neural stem cells, occurs predominantly in the subventricular zone and the hippocampal dentate gyrus, regions critical for memory and learning. This process involves proliferation, migration, differentiation, and integration of newborn neurons into existing circuits. Neuronal survival ensures that these newly generated cells persist and contribute to functional networks, a balance regulated by trophic factors, signaling pathways, and environmental cues [50], although most studies examining how marine-derived compounds influence these processes remain limited to in vitro and animal models.

Growth factors such as brain-derived neurotrophic factor (BDNF) and nerve growth factor (NGF) play pivotal roles in promoting both neurogenesis and neuronal survival. BDNF enhances synaptic plasticity and supports the maturation of newborn neurons, while NGF regulates neuronal differentiation and prevents apoptosis. These trophic signals converge on intracellular cascades such as the PI3K/Akt and MAPK/ERK pathways, which promote cell survival and growth. Conversely, dysregulation of these pathways contributes to neurodegenerative conditions, highlighting their therapeutic relevance [51], although most studies examining how marine-derived compounds modulate these signaling mechanisms remain restricted to in vitro and animal models.

Inflammation and oxidative stress exert profound effects on neurogenesis. Chronic neuroinflammation, mediated by activated microglia and pro-inflammatory cytokines, impairs stem cell proliferation and reduces neuronal survival. Similarly, excessive ROS damage DNA and proteins, leading to the apoptosis of vulnerable neurons. Antioxidant and anti-inflammatory mechanisms, including Nrf2 activation and NF-κB inhibition, are therefore essential for maintaining a permissive environment for neurogenesis [52], although most studies evaluating how marine-derived compounds modulate these pathways remain restricted to in vitro and animal models.

Additional evidence comes from studies on extracts of the edible red alga *Gracilariopsis chorda*, which has shown protective effects against hypoxia/reoxygenation-induced oxidative stress in cultured hippocampal neurons. These extracts reduced ROS accumulation, preserved mitochondrial function, and improved neuronal viability in vitro, supporting the potential relevance of red algal metabolites for neuroprotection, although their efficacy in humans remains unverified [21].

Marine-derived bio-actives have emerged as potential modulators of these processes. Compounds such as fucoxanthin, phlorotannins, and omega-3 fatty acids modulate BDNF signaling, reduce oxidative stress, and influence inflammatory pathways in experimental models. Omega-3 fatty acids, particularly docosahexaenoic acid (DHA), are incorporated into neuronal membranes, improving fluidity and resilience and promoting neurogenesis in the hippocampus in preclinical studies [36]. Phlorotannins from brown algae attenuate apoptosis and support neuronal survival through antioxidant and anti-inflammatory mechanisms in vitro and in vivo, suggesting possible nutraceutical relevance, although their efficacy in humans remains unproven [30].

Together, neurogenesis and neuronal survival represent dynamic processes that integrate molecular signaling, environmental modulation, and dietary influences. Their modulation by marine bio-actives has been demonstrated primarily in in vitro and animal studies, suggesting a potential role for ocean-derived compounds in supporting brain health and resilience. However, the translational relevance of these findings remains to be established, and further clinical research is required before marine-based interventions can be considered viable neuroprotective strategies.

Mitochondria are central regulators of neuronal energy metabolism, calcium homeostasis, and apoptotic signaling, making their function critical for brain health and resilience. Neurons, with their high energy demands, rely heavily on oxidative phosphorylation to sustain synaptic transmission and plasticity. Disruption of mitochondrial function leads to impaired ATP production, excessive generation of ROS, and activation of cell death pathways, all of which contribute to neurodegenerative processes [53]. Most studies examining how marine-derived compounds influence mitochondrial dynamics and bioenergetics have been conducted in vitro or in animal models, and their relevance for human neurodegeneration remains to be clarified.

Mitochondrial dynamics—including fusion, fission, and mitophagy—play essential roles in maintaining neuronal integrity. Fusion allows for the mixing of mitochondrial contents to dilute damage, while fission facilitates the removal of dysfunctional organelles. Mitophagy, the selective autophagic clearance of damaged mitochondria, prevents the accumulation of defective organelles that would otherwise exacerbate oxidative stress. Dysregulation of these processes has been implicated in Alzheimer’s disease, Parkinson’s disease, and amyotrophic lateral sclerosis (ALS), underscoring their importance in neuronal survival [54], although most studies examining how marine-derived compounds influence mitochondrial dynamics remain restricted to in vitro and animal models.

Calcium buffering is another critical mitochondrial function. Neuronal activity requires precise regulation of intracellular calcium, and mitochondria act as reservoirs that prevent excitotoxicity. Excessive calcium uptake, however, can trigger mitochondrial permeability transition pore (mPTP) opening, leading to loss of membrane potential and the initiation of apoptosis. This mechanism links mitochondrial dysfunction directly to neuronal death in pathological conditions. DHA enhances BDNF expression and synaptic plasticity in preclinical studies, while EPA reduces mitochondrial ROS and improves membrane stability in vitro and in vivo. Fucosterol, a major algal sterol, activates PI3K/Akt and ERK signaling pathways and supports neuronal survival under oxidative stress in experimental models [55], although its relevance for human neuroprotection remains to be established.

Marine-derived bio-actives have shown promise in modulating mitochondrial function in experimental models. Fucoxanthin, a carotenoid from brown algae, enhances mitochondrial biogenesis and protects against oxidative stress by activating the Nrf2/ARE pathway in vitro and in vivo. Phlorotannins, polyphenolic compounds unique to marine algae, reduce mitochondrial ROS production and stabilize membrane potential, thereby supporting neuronal survival in preclinical studies. Omega-3 fatty acids, particularly DHA, are incorporated into mitochondrial membranes, improving fluidity and electron transport efficiency while reducing susceptibility to oxidative damage [36]. These findings suggest that marine nutraceuticals may help restore mitochondrial function, although their neuroprotective efficacy in humans remains to be demonstrated.

Overall, mitochondrial function represents a nexus between energy metabolism, oxidative stress, and cell survival. Its modulation by marine bio-actives has shown promise in experimental models, aligning with the broader vision of marine medicine to harness ocean-derived compounds for neurological health. However, several studies report minimal or no neuroprotective effects depending on extraction method, species, and experimental conditions. Negative or inconclusive findings are often underreported, which may bias interpretation and highlights the need for standardized methodologies and rigorous validation in future research.

## 5. Seaweed Extracts in Neurodegenerative Disorders

Seaweed extracts (see Table 3) are increasingly investigated for their preclinical effects relevant to neurodegenerative disorders. However, these findings do not yet support therapeutic use in humans. No clinical trials have demonstrated disease-modifying effects in Alzheimer’s, Parkinson’s, or other neurodegenerative conditions.

Rich in polysaccharides, phlorotannins, carotenoids, and sterols, these marine-derived molecules exert antioxidant, anti-inflammatory, and anti-apoptotic effects that directly counteract pathological processes such as oxidative stress, mitochondrial dysfunction, and protein aggregation. Recent studies highlight the neuroprotective potential of fucoidan, laminarin, ulvan and porphyran in modulating immune responses and reducing neuronal damage, while phlorotannins and fucoxanthin demonstrate strong activity against amyloid-β toxicity and dopaminergic cell loss [31,56]. Moreover, seaweed polyphenols have been shown to enhance synaptic plasticity and protect against excitotoxicity, suggesting their relevance in Alzheimer’s, Parkinson’s, and other age-related neurodegenerative conditions. Although most evidence derives from preclinical models, the accumulating data support the integration of seaweed extracts into nutraceuticals and functional foods as complementary strategies for neuroprotection, while calling for rigorous clinical trials to validate their efficacy and safety in human populations [57].

**Table 3:** Seaweed Extracts in Neurodegenerative Disorders.

Disorder	Seaweed/Extract	Species Name	Reported Effect	Mechanism	Evidence Level	Refs.
Alzheimer’s disease	Fucoxanthin, phlorotannins	*Undaria pinnatifida*, *Ecklonia cava*	Reduced amyloid-beta aggregation, improved cognition	Antioxidant, anti-amyloid	Preclinical	[7,58]
Parkinson’s disease	Fucoidan, omega-3 fatty acids	*Fucus vesiculosus*, *Ulva lactuca*	Protection of dopaminergic neurons	Anti-inflammatory, mitochondrial support	Animal models	[4,10]
Multiple sclerosis	Polysaccharides	*Chondrus crispus*	Reduced neuroinflammation	Immunomodulation	Preclinical	[9]
Depression	Phlorotannins	*Ecklonia cava*	Antidepressant-like effects	Modulation of monoamine neurotransmitters	Rodent models	[59]

Note: All reported effects derive from preclinical studies or animal models; no clinical evidence of disease modification exists.

Fucoidan and phlorotannins currently have the strongest preclinical support, with multiple mechanistic and in vivo studies. Carotenoids such as fucoxanthin show promising but still limited validation, while peptides, sterols, and minor metabolites remain underexplored and supported mainly by preliminary findings. Differentiating between well-supported and emerging evidence is essential to avoid overstating the maturity of the field [17].

Phlorotannins from *Ecklonia cava* and *Eisenia bicyclis* inhibit Aβ fibrillogenesis, reduce oxidative stress-induced tau phosphorylation, and downregulate GSK-3β and CDK5 activity in vitro and in vivo. These mechanisms suggest a potential role in modulating amyloidogenesis and tau pathology in preclinical models [4], although their relevance for human neurodegeneration remains to be established.

### 5.1. Alzheimer’s Disease: Amyloid-Beta Aggregation, Tau Pathology

Alzheimer’s disease (AD) is characterized by the extracellular accumulation of amyloid-β (Aβ) plaques and the intracellular aggregation of hyperphosphorylated tau protein, both of which disrupt neuronal communication and contribute to progressive cognitive decline. Seaweed extracts have emerged as potential sources of bioactive compounds capable of modulating these pathological processes in preclinical models. Sulfated polysaccharides such as fucoidan, derived from brown algae, inhibit Aβ fibril formation and destabilize preformed aggregates in vitro and in vivo, thereby reducing plaque burden in experimental systems. Fucoidan also enhances autophagic clearance of misfolded proteins, supporting neuronal survival under pathological conditions [25].

Phlorotannins, polyphenolic compounds unique to seaweed, exert antioxidant and anti-amyloid activities by scavenging ROS and directly interfering with Aβ aggregation. These compounds protect neurons from Aβ-induced oxidative stress in preclinical studies, suggesting possible relevance for AD-related mechanisms, although their efficacy in humans remains unproven [60].

Tau pathology, marked by hyperphosphorylation and subsequent formation of neurofibrillary tangles, is another critical target explored in studies of seaweed-derived bio-actives. Fucoxanthin, a carotenoid abundant in brown algae, modulates kinase activity—particularly glycogen synthase kinase 3β (GSK-3β) and cyclin-dependent kinase 5 (CDK5)—which are central regulators of tau phosphorylation. By attenuating these pathways, fucoxanthin reduces tau misfolding and stabilizes microtubule dynamics in preclinical models [61]. Seaweed-derived phytosterols may further influence tau regulation by modulating cholesterol metabolism in neuronal membranes, thereby affecting tau stability and reducing aggregation in experimental systems, although their relevance for human disease remains to be determined.

The therapeutic potential of seaweed extracts lies in their multi-targeted mechanisms. Beyond direct effects on Aβ and tau, these compounds exert anti-inflammatory and antioxidant actions that may help interrupt the cycle of oxidative stress and neuroinflammation contributing to AD pathology. Marine polysaccharides also enhance neuronal resilience by promoting autophagy and supporting synaptic plasticity in experimental models. Preclinical studies, including supplementation of *Sargassum fusiforme* (Phaeophyceae) extracts in transgenic AD mouse models, have reported reductions in Aβ deposition and improvements in cognitive performance [62], although these findings remain restricted to animal systems and their translational relevance is uncertain.

Some studies have failed to reproduce these effects, and variability in extract composition, species, and extraction methods complicates interpretation, underscoring the need for standardized protocols and rigorous validation.

### 5.2. Parkinson’s Disease: Dopaminergic Neuron Protection

Parkinson’s disease (PD) is a progressive neurodegenerative disorder primarily characterized by the loss of dopaminergic neurons in the substantia nigra pars compacta, leading to motor and non-motor symptoms. The degeneration of these neurons is driven by multiple pathological processes, including oxidative stress, mitochondrial dysfunction, neuroinflammation, and aggregation of α-synuclein. Seaweed extracts have emerged as potential sources of bioactive compounds capable of modulating these mechanisms in preclinical models.

Brown algae-derived polysaccharides such as fucoidan reduce oxidative stress and inhibit apoptotic pathways in vitro and in vivo. Fucoidan upregulates antioxidant enzymes, stabilizes mitochondrial function, and suppresses pro-inflammatory cytokine release, thereby creating a more favorable environment for dopaminergic neuron survival in experimental systems [26]. Similarly, phlorotannins, unique polyphenols from seaweed, act as free radical scavengers and modulate intracellular signaling cascades such as the PI3K/Akt pathway, which supports neuronal survival and resilience against oxidative insults in preclinical studies [30].

Marine carotenoids, particularly fucoxanthin, contribute to dopaminergic protection in preclinical models by enhancing mitochondrial biogenesis and reducing neuroinflammation. Fucoxanthin inhibits microglial activation and attenuates the release of pro-inflammatory mediators in vitro and in vivo, thereby limiting secondary damage to dopaminergic neurons [7]. Omega-3 fatty acids from marine sources, especially DHA, further support dopaminergic survival by improving membrane fluidity, modulating synaptic plasticity, and reducing α-synuclein aggregation in experimental systems [36], although their relevance for human Parkinson’s disease remains to be established.

The combined antioxidant, anti-inflammatory, and mitochondrial-stabilizing properties of seaweed extracts highlight their potential relevance for dopaminergic protection in preclinical models of PD. By targeting multiple pathological mechanisms simultaneously, these compounds may help limit dopaminergic degeneration and support neuronal resilience in experimental systems. Although most evidence remains restricted to in vitro and animal studies, the exploration of seaweed bio-actives as components of nutraceutical or adjunct strategies is scientifically promising, while rigorous clinical trials are required to determine their efficacy and safety in humans.

### 5.3. Other Conditions: Multiple Sclerosis, Epilepsy, Depression

Beyond Alzheimer’s and Parkinson’s disease, seaweed extracts have demonstrated potential in modulating the pathophysiology of other neurological conditions, including multiple sclerosis (MS), epilepsy, and depression. These disorders, though distinct in etiology, share common mechanisms such as oxidative stress, neuroinflammation, and impaired neurotransmission, all of which can be targeted by marine bio-actives.

In MS, an autoimmune demyelinating disease, neuroinflammation and oxidative damage drives progressive neuronal dysfunction. Sulfated polysaccharides such as fucoidan and carrageenan have been reported to attenuate inflammatory cascades by suppressing pro-inflammatory cytokines and modulating immune cell activity. Fucoidan, in particular, reduces microglial activation and enhances remyelination processes, suggesting its potential as a supportive therapy in MS [16]. Phlorotannins from brown algae further contribute by scavenging ROS and stabilizing mitochondrial function, thereby protecting oligodendrocytes and promoting neuronal survival [30].

Epilepsy, characterized by recurrent seizures due to abnormal neuronal excitability, may also benefit from seaweed-derived compounds. Phlorotannins and fucoxanthin exhibit GABAergic modulatory effects, enhancing inhibitory neurotransmission and reducing excitotoxicity. Experimental studies have shown that fucoxanthin can attenuate seizure severity by stabilizing neuronal membranes and reducing oxidative stress [7]. Moreover, omega-3 fatty acids from marine sources improve synaptic plasticity and modulate ion channel activity, contributing to seizure control and neuroprotection [36].

Depression, a multifactorial disorder involving dysregulation of monoaminergic neurotransmission, neuroinflammation, and impaired neurogenesis, has also been explored in studies of seaweed-derived bio-actives. Fucoxanthin upregulates BDNF and modulates serotonergic signaling in preclinical models, effects associated with improved mood-related and cognitive outcomes in experimental systems. Phlorotannins exert antidepressant-like actions by reducing oxidative stress and inflammatory markers while omega-3 fatty acids enhance synaptic plasticity and hippocampal neurogenesis, mechanisms central to resilience against depressive symptoms [60].

Taken together, current evidence suggests that seaweed extracts act on convergent pathways, including antioxidant defense, anti-inflammatory signaling, neurotransmitter modulation, and neurotrophic support, which are relevant across diverse neurological disorders. However, as most findings derive from in vitro and animal studies, clinical validation remains limited. These results support continued investigation of marine bio-actives as potential contributors to neuronal health and resilience, while underscoring the need for rigorous human studies before their integration into therapeutic or preventive strategies.

## 6. Preclinical and Clinical Evidence

### 6.1. In Vitro Studies

In vitro studies have provided important insights into the neuroprotective potential of seaweed-derived compounds, particularly phlorotannins, fucoidans, fucoxanthin, and omega-3 fractions. These bio-actives mitigate oxidative damage, stabilize mitochondrial function, and modulate pro-survival signaling pathways in human and rodent neuronal cell models. For example, phlorotannins unique to brown macroalgae attenuate ROS, preserve mitochondrial membrane potential, and suppress caspase-3 activation in vitro, thereby reducing amyloid-β- and dopamine-induced toxicity in neuronal cultures [11,30].

Human neuroblastoma SH-SY5Y cells are frequently employed to evaluate the effects of seaweed extracts under oxidative and dopaminergic stress. In these models, whole seaweed extracts help maintain cell viability, preserve mitochondrial function, and reduce apoptotic signaling, supporting a mitochondria-centered mechanism of protection in vitro that is relevant to PD-related pathways [63]. Complementary evidence from broader cellular stress paradigms shows that seaweed polyphenols and carotenoids reinforce antioxidant defenses and limit excitotoxic cascades in neuronal cultures, although their translational relevance remains uncertain [56].

Mechanistically, marine bio-actives act on convergent nodes of neurodegeneration. Phlorotannins and fucoxanthin modulate Nrf2/ARE signaling, inhibit NF-κB activity, and influence aggregation-prone proteins, while sulfated polysaccharides such as fucoidan enhance autophagic flux and neurotrophic support. These multimodal actions, consistently observed across in vitro platforms, provide a rationale for further in vivo validation, although translation to early phase clinical studies will require more robust and reproducible evidence [31].

### 6.2. Animal Models

Animal models have been indispensable for evaluating the neuroprotective potential of seaweed-derived bio-actives and for extending observations from in vitro systems into whole-organism contexts. Rodent models of AD, PD, epilepsy, and depression have been particularly informative, showing that seaweed extracts can modulate convergent mechanisms such as oxidative stress, neuroinflammation, protein aggregation, and synaptic dysfunction in vivo. These findings strengthen the preclinical foundation for further investigation, although translation to human applications remains uncertain.

In AD models, supplementation with *Sargassum fusiforme* extracts in APP/PS1 transgenic mice has been reported to reduce amyloid-β deposition, attenuate tau hyperphosphorylation, and improve performance in behavioral assays [62]. Fucoidan administration in similar models enhances autophagic clearance of misfolded proteins and reduces neuroinflammation, supporting its involvement in proteostasis and neuronal survival in vivo [59]. Phlorotannins have likewise been shown to improve memory-related performance in rodent models by modulating cholinergic neurotransmission and reducing oxidative stress [30], although these effects remain restricted to preclinical systems.

In PD models, fucoxanthin and fucoidan demonstrated dopaminergic neuroprotection. Fucoxanthin reduced microglial activation and preserved dopaminergic neurons in the substantia nigra of MPTP-treated mice, while fucoidan attenuated oxidative stress and stabilized mitochondrial function, thereby improving motor performance [7,26]. These findings highlight the multi-targeted actions of seaweed bio-actives in protecting vulnerable neuronal populations. Most available data involve surrogate biomarkers or subjective outcomes, which cannot be interpreted as evidence of neuroprotection.

Other neurological conditions have also been investigated in animal models. In experimental autoimmune encephalomyelitis (EAE), a model of MS, fucoidan reduces demyelination and suppresses pro-inflammatory cytokine release in vivo, indicating immunomodulatory activity in preclinical systems [16]. In rodent models of epilepsy, fucoxanthin attenuates seizure severity by modulating GABAergic neurotransmission and reducing oxidative stress [7]. Depression models show that fucoxanthin and phlorotannins upregulate BDNF and modulate serotonergic signaling, effects associated with improved behavioral outcomes in these experimental paradigms [60]. However, these findings remain restricted to animal studies, and their relevance for human neurological or psychiatric conditions has yet to be established.

Collectively, animal studies indicate that seaweed extracts can modulate pathological processes across diverse models of neurodegeneration and psychiatric disease. Their ability to act on multiple pathways, including oxidative stress, inflammation, mitochondrial dysfunction, and protein aggregation, supports continued preclinical investigation. However, these findings should not be interpreted as evidence of therapeutic efficacy, and their relevance for nutraceutical or adjunct applications remains uncertain. Further progress will require standardized extracts, consistent dosing strategies, and rigorous study designs to improve reproducibility and translational value.

Despite these encouraging observations, rodent models of AD and PD have limited predictive validity. Reductions in amyloid burden or improvements in motor behavior do not reliably translate into clinical benefit in humans, underscoring the need for cautious interpretation of preclinical outcomes.

### 6.3. Human Trials

Human clinical evidence remains extremely limited, and no study to date has demonstrated direct effects of seaweed-derived compounds on neurodegenerative pathology. Most available data originate from small-scale dietary interventions, pilot supplementation trials, or epidemiological observations in populations with high habitual seaweed consumption. These studies indicate that seaweed bio-actives may improve systemic antioxidant status and modulate inflammatory markers, with some reports suggesting indirect benefits for cognitive performance. However, these outcomes are nonspecific and cannot be interpreted as evidence of disease-modifying effects in neurodegeneration.

Dietary intake of brown algae such as *Undaria pinnatifida* and *Sargassum fusiforme* has been associated with reduced risk of cognitive decline in East Asian cohorts, where seaweed consumption is culturally embedded. Observational studies report correlations between regular seaweed intake and improved memory scores, although these associations are indirect and cannot establish a protective effect against age-related neurodegeneration [64]. Pilot supplementation trials have also reported improvements in mood, sleep quality, and stress resilience following seaweed extract intake, outcomes relevant to depression and anxiety. However, these findings derive from small studies and have not yet been validated in large, randomized controlled trials [11].

Specific bioactive compounds from seaweed have also been evaluated in human studies. Fucoxanthin, a carotenoid abundant in brown algae, has been investigated for its antioxidant and anti-inflammatory properties, with some trials reporting reductions in systemic oxidative stress markers and improvements in lipid metabolism, effects that may be indirectly relevant to neurodegenerative pathways [32]. Astaxanthin supplementation has shown preliminary benefits in small clinical studies, including modest improvements in cognitive performance and reduced fatigue in healthy adults, although these findings remain exploratory [33]. Omega-3 fatty acids derived from marine algae, particularly DHA, have extensive human trial evidence supporting their role in cognitive health, memory, and general neurological resilience, positioning seaweed as a sustainable source of these essential lipids. However, these outcomes do not constitute evidence of disease-modifying effects in neurodegeneration [65].

Despite these promising findings, important limitations remain. Most studies are small, short-term, and rely on surrogate biomarkers rather than direct neurological outcomes. Standardization of seaweed extracts, clarification of bioavailability, and long-term safety assessments represent critical challenges for any future clinical development. While epidemiological observations, pilot trials, and mechanistic insights collectively support the continued investigation of seaweed-derived compounds, these data do not yet provide a sufficient basis for disease-specific clinical trials in AD, PD, or related disorders. Larger, well-controlled studies will be required to determine whether these bio-actives exert meaningful effects in humans.

## 7. Challenges and Limitations

### 7.1. Bioavailability and Pharmacokinetics

Sulfated polysaccharides such as fucoidan and carrageenan exhibit extremely low intestinal absorption, undergo extensive metabolism by gut microbiota, and show minimal systemic bioavailability. To date, no evidence supports their penetration into the central nervous system. Phlorotannins also display rapid hepatic metabolism, extensive glucuronidation and sulfation, and low oral bioavailability in both rodent and human studies, substantially limiting their capacity to reach concentrations relevant for direct neurobiological effects [3,4,16,19].

To date, no clinical trials have demonstrated neuroprotective effects of seaweed-derived compounds in Alzheimer’s or Parkinson’s disease. Existing human studies primarily assess systemic antioxidant status, metabolic outcomes, or cognitive fatigue, without direct evaluation of neurodegenerative endpoints. Despite encouraging preclinical findings, a major challenge in translating seaweed-derived bio-actives into potential neuroprotective interventions lies in their bioavailability and pharmacokinetics. Many compounds isolated from marine algae, including phlorotannins, fucoidan, fucoxanthin, and sulfated polysaccharides, exhibit poor oral absorption, rapid metabolism, and limited capacity to cross the blood–brain barrier (BBB). These pharmacokinetic constraints substantially limit their in vivo activity, even when robust effects are observed in vitro [16,66].

Polyphenols such as phlorotannins are highly hydrophilic and undergo extensive first-pass metabolism, resulting in low systemic concentrations after oral administration. Fucoidan, a high-molecular-weight sulfated polysaccharide, also faces substantial barriers to intestinal absorption due to its size and polarity, leading to minimal systemic exposure and negligible central nervous system availability [16]. Carotenoids such as fucoxanthin are lipophilic and require incorporation into micelles for efficient absorption, but their conversion into active metabolites (e.g., amarouciaxanthin A) varies widely among individuals, adding further complexity to pharmacokinetic predictability [7].

Crossing the BBB represents an additional barrier. While small lipophilic molecules such as Omega-3 fatty acids (DHA, EPA) can integrate into neuronal membranes, larger polysaccharides and polyphenols generally fail to reach measurable concentrations in the brain. Strategies including nanoparticle encapsulation, liposomal delivery, and conjugation with carrier molecules are being explored to enhance brain penetration and prolong systemic half-life, although these approaches remain experimental and have not yet demonstrated efficacy in neurodegenerative contexts [31,66].

Another limitation is the variability in digestive stability and metabolism. Seaweed bio-actives are subject to enzymatic degradation in the gastrointestinal tract, and their metabolites may differ substantially in activity from the parent compounds. This variability complicates dose standardization and reduces reproducibility across studies. In addition, interindividual differences in gut microbiota composition influence the breakdown and absorption of seaweed polysaccharides, introducing further complexity into their pharmacokinetic behavior [31,56].

Overall, although seaweed-derived compounds show promising biological activity in preclinical models, their limited bioavailability and unpredictable pharmacokinetics remain major obstacles to any potential clinical translation. Addressing these challenges will require advanced formulation strategies, standardized extraction protocols, and rigorous pharmacokinetic evaluation in human studies. At present, these limitations substantially reduce the likelihood that orally consumed seaweed extracts achieve concentrations in the central nervous system sufficient to exert direct neurobiological effects [67].

### 7.2. Standardization of Extracts

**Figure 3:**
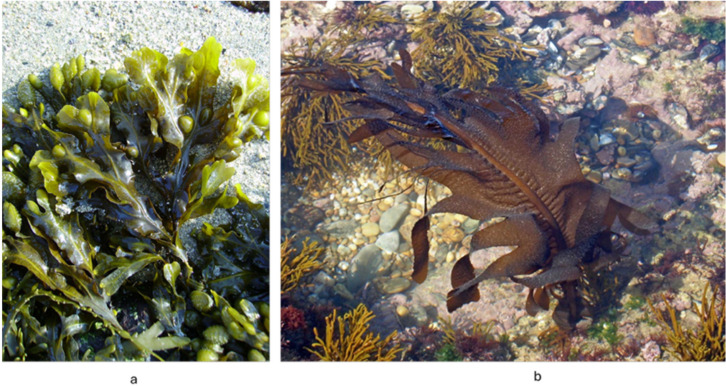
*Fucus vesiculosus* (**a**), *Undaria pinnatifida* (**b**).

A major limitation in advancing seaweed-derived compounds toward clinical evaluation is the lack of standardized extracts. Seaweed bio-actives, including phlorotannins, fucoidan, laminarin, ulvan, and carotenoids such as fucoxanthin, show substantial variability in chemical composition depending on species, geographic origin, seasonality, and extraction procedures. This heterogeneity complicates reproducibility across studies and prevents the establishment of consistent pharmacological profiles. For example, fucoidan isolated from *Fucus vesiculosus* (Figure 3a) differs markedly in sulfation pattern and molecular weight from fucoidan obtained from *Undaria pinnatifida* (Figure 3b), resulting in divergent biological activities [16,28].

Extraction techniques further contribute to variability. Conventional solvent extraction, enzymatic hydrolysis, and advanced methods such as supercritical fluid extraction or microwave-assisted extraction yield different concentrations and structural variants of bio-actives. These methodological differences influence antioxidant capacity, anti-inflammatory activity, and reported neurobiological effects, making cross-study comparisons difficult [66]. In addition, the absence of standardized reference materials and validated analytical protocols for quantifying seaweed metabolites limits the ability to relate administered doses to biological outcomes in a consistent and reproducible manner [38].

Another challenge is the stability of extracts during storage and processing. Polyphenols and carotenoids are prone to oxidation, while polysaccharides may undergo depolymerization, altering their biological activity. Without standardized stabilization and formulation strategies, extract potency may decline substantially before reaching experimental or clinical evaluation. This issue highlights the need for harmonized guidelines on extraction, purification, and storage conditions to ensure reproducibility and comparability across studies [35,67].

To address these limitations, researchers advocate for the development of quality-control frameworks similar to those used in herbal medicine, including chemical fingerprinting, metabolomic profiling, and bioassay-guided standardization. Establishing international standards for seaweed extract preparation (Table 4) would improve reproducibility and support regulatory evaluation. Ultimately, standardized extracts are essential for assessing the biological activity and safety of marine bio-actives, but they are only a first step toward determining whether these compounds hold therapeutic relevance for neurodegenerative or psychiatric disorders [11,31].

### 7.3. Safety and Toxicity Concerns

Although seaweed-derived compounds are generally regarded as safe and have a long history of dietary use, their safety and toxicity profiles require careful evaluation before any clinical application. For most seaweed-derived polysaccharides and polyphenols, measurable concentrations in brain tissue have not been demonstrated in humans.

Seaweed can also accumulate environmental contaminants such as heavy metals (arsenic, cadmium, lead) and excessive iodine, which may pose health risks if consumed in large amounts or without appropriate monitoring. Chronic exposure to inorganic arsenic, for example, has been associated with neurotoxicity and carcinogenicity, underscoring the need for strict quality-control procedures during extract preparation [71].

One major concern is the accumulation of heavy metals such as arsenic, cadmium, and lead, which can vary substantially depending on the geographic origin and harvesting conditions of seaweed. Chronic exposure to inorganic arsenic, in particular, has been associated with neurotoxicity and carcinogenicity, raising important questions about the safety of long-term supplementation [71]. Regulatory agencies therefore emphasize the need for strict monitoring of contaminant levels in commercial extracts to ensure consumer safety.

Dose-dependent effects also represent an important challenge. While fucoidan, laminarin, and ulvan are generally well-tolerated, high concentrations may interfere with coagulation pathways or immune responses. Similarly, phlorotannins, although potent antioxidants, can exhibit pro-oxidant activity at excessive doses, underscoring the need to establish safe intake thresholds [72,73]. Digestive tolerance is another concern, as carrageenan and agar from red algae have been associated with gastrointestinal discomfort in sensitive individuals [63].

Seasonal and geographic variability strongly influence iodine and heavy-metal content, which may exceed the recommended intake levels. These factors must be considered when evaluating safety and reproducibility.

Variability in metabolism adds further complexity. Seaweed polysaccharides are broken down differently depending on gut microbiota composition, which can lead to unpredictable systemic effects. This interindividual variability complicates dose standardization and reduces reproducibility in clinical settings [31].

Finally, the lack of standardized toxicological studies remains a major limitation. Most safety data derive from dietary observations rather than controlled trials. Comprehensive toxicokinetic profiling—including absorption, distribution, metabolism, and excretion (ADME)—is essential to determine safe dosage ranges and long-term effects. Without such data, extrapolation from preclinical models to humans remains highly uncertain [37].

### 7.4. Regulatory Aspects

The translation of seaweed-derived bio-actives into clinical applications is constrained not only by scientific challenges but also by regulatory frameworks that govern their classification, safety, and commercialization. Seaweed extracts occupy a complex regulatory space, often spanning categories such as food, dietary supplements, nutraceuticals, and pharmaceuticals. This ambiguity complicates approval pathways and limits the development of standardized guidelines for potential neuroprotective applications.

In the European Union (EU), seaweed extracts are generally regulated under the Novel Food Regulation (EU 2015/2283), which requires safety assessments and authorization before commercialization if the product was not widely consumed prior to 1997. Extracts intended for therapeutic use must comply with European Medicines Agency (EMA) standards, including rigorous toxicological and clinical evaluation [37]. In the United States, seaweed-derived compounds marketed as dietary supplements fall under the Dietary Supplement Health and Education Act (DSHEA 1994) [74], which permits commercialization without prior FDA approval but restricts disease-related claims. Products intended for pharmaceutical use must undergo the full FDA approval process, including Investigational New Drug (IND) applications and clinical trials [28].

Globally, regulatory approaches vary. In Japan and Korea, seaweed is widely accepted as a traditional food, and extracts such as fucoidan and laminarin are marketed as functional foods with comparatively fewer regulatory barriers. In contrast, China has introduced stricter requirements for functional foods and nutraceuticals, demanding evidence of safety, efficacy, and quality control [35]. These differences highlight the need for harmonized international standards to support consistent quality assurance and facilitate responsible commercialization.

Another regulatory challenge is the standardization of extracts. Agencies emphasize the importance of validated analytical methods, chemical fingerprinting, and batch-to-batch consistency to ensure reproducibility. Without standardized protocols, variability in composition undermines both safety and efficacy assessments, delaying regulatory evaluation [38]. Furthermore, labeling requirements regarding iodine content, heavy-metal contamination, and allergenicity are increasingly enforced, reflecting growing consumer safety concerns [72].

In summary, the regulatory landscape for seaweed-derived bio-actives is fragmented, with substantial differences across regions. Harmonized international guidelines, standardized extraction protocols, and validated safety assessments will be essential to ensure product quality and safety. These measures are necessary prerequisites for any future evaluation of seaweed-derived compounds in neurodegenerative contexts, but they do not guarantee clinical translation in themselves.

## 8. Future Perspectives

### 8.1. Potential for Nutraceuticals and Functional Foods

Seaweed-derived bio-actives have attracted growing interest for potential use in nutraceuticals and functional foods, offering a sustainable source of compounds with relevance to general brain health. Their composition, rich in polysaccharides, polyphenols, carotenoids, sterols, and Omega-3 fatty acids, supports multiple biological pathways implicated in neurological function. Unlike single-target pharmaceuticals, seaweed compounds may influence several processes simultaneously, including oxidative stress, neuroinflammation, mitochondrial function, and protein homeostasis, which has motivated their exploration in functional-food formulations.

Several seaweed extracts are already incorporated into commercial nutraceuticals. Fucoidan from *Fucus vesiculosus* and *Undaria pinnatifida* is marketed for general immune and anti-inflammatory support, while phlorotannin-rich extracts from Ascophyllum nodosum are included in antioxidant formulations [28]. Fucoxanthin, a carotenoid from brown algae, has attracted interest for its reported effects on metabolic regulation, although evidence for neurobiological benefits remains preliminary and limited to small pilot studies [32]. Ulvan from *Ulva lactuca* and porphyran from Porphyra/*Pyropia* spp. are also being explored as functional-food ingredients due to their potential immunomodulatory and gut–brain axis interactions [38].

The functional-food sector offers a practical route for incorporating seaweed-derived compounds into everyday diets through fortified beverages, snack bars, capsules, or fermented products. This approach avoids some of the regulatory requirements associated with pharmaceuticals, although it does not replace the need for rigorous safety and efficacy evaluation. Growing consumer interest in plant-based, sustainable, and natural products has increased market attention toward seaweed-derived ingredients, but their positioning within the nutraceutical sector remains dependent on standardized characterization and evidence-based assessment of their biological effects [35].

However, several challenges remain. Standardization of extracts, optimization of bioavailability, and long-term safety assessments are essential to support reliable use in nutraceutical and functional-food contexts. Advances in encapsulation technologies, emulsions, and co-formulation with lipids are being investigated to improve absorption and stability [37]. Collaborative efforts between academia, industry, and regulatory agencies will be necessary to establish evidence-based health claims and ensure that seaweed-derived ingredients meet the quality and safety requirements for mainstream functional-food markets. Seaweed-derived compounds represent an emerging area of interest in nutraceuticals and functional foods, combining scientific exploration with sustainability and consumer appeal. Their incorporation into daily diets may offer accessible ways to support general health, but their relevance to neurobiological outcomes remains to be demonstrated through rigorous human studies.

### 8.2. Integration into Marine Medicine and Personalized Therapies

The integration of seaweed-derived bio-actives into marine medicine and personalized therapies is an emerging area of translational research that seeks to connect ecological sustainability with individualized healthcare approaches. Marine medicine highlights the potential relevance of ocean-derived compounds, and seaweed extracts, rich in polysaccharides, polyphenols, carotenoids, and sterols, are being explored for their possible contributions to systemic and neurological health. Their multi-targeted biological activities, including antioxidant, anti-inflammatory, immunomodulatory, and metabolic effects, align with several pathways implicated in neurodegenerative and psychiatric conditions, although their clinical significance remains to be established.

Personalized-medicine frameworks may further shape the investigation of seaweed bio-actives. Advances in nutrigenomics and metabolomics enable dietary interventions to be tailored according to genetic background, metabolic status, and gut-microbiota composition. For example, individuals with specific polymorphisms affecting oxidative-stress pathways might respond differently to phlorotannin-rich extracts, while those with inflammatory phenotypes could show distinct responses to fucoidan or ulvan supplementation [56]. Integrating seaweed-derived compounds into personalized-nutrition platforms may therefore help refine hypotheses about targeted dietary strategies, but their neurobiological relevance requires validation in controlled human studies.

**Figure 4:**
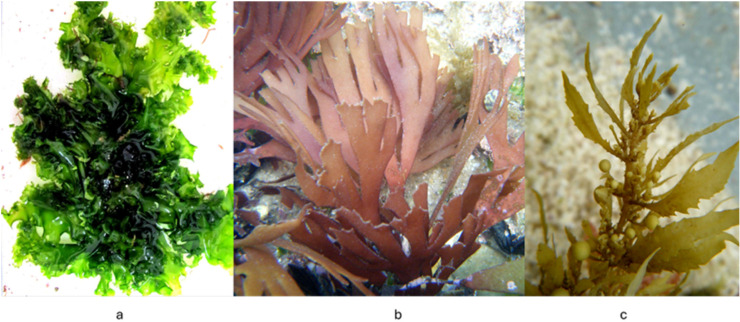
*Ulva* (**a**), *Gracilaria* (**b**), and *Sargassum* (**c**).

Marine medicine also emphasizes sustainability and responsible resource management, ensuring that the use of seaweed for therapeutic investigation does not compromise marine ecosystems. Cultivation of *Ulva* (Chlorophyta) (Figure 4a), *Gracilaria* (Rhodophyta) (Figure 4b), and *Sargassum* (Phaeophyceae) (Figure 4c) in controlled aquaculture systems provide standardized biomass for extract production, reducing variability and improving traceability [28]. This approach supports ecological conservation and enhances reproducibility—both central principles of marine-medicine frameworks.

Emerging technologies such as nanoencapsulation, liposomal delivery, and biopolymer conjugation are being explored to improve the bioavailability and dosing precision of seaweed-derived bio-actives. These approaches aim to enhance pharmacokinetic consistency and may support more targeted delivery, although their relevance for neural tissues remains to be demonstrated [38]. Digital-health platforms and AI-driven analytics are also being investigated as tools to monitor individual responses to seaweed-based interventions, potentially enabling adaptive adjustments and more personalized nutritional strategies [35].

The convergence of marine-medicine concepts with personalized-therapy frameworks has stimulated interest in seaweed-derived compounds as sustainable and adaptable resources for future health applications. By integrating ecological stewardship, emerging biotechnologies, and individualized healthcare approaches, seaweed bio-actives may progress from traditional dietary components to candidates for more targeted nutritional interventions. However, their potential relevance for neurological or systemic health requires rigorous validation in controlled human studies before any therapeutic implications can be established.

### 8.3. Emerging Technologies (Nano-Delivery, Biotechnological Production)

The development of seaweed-derived bio-actives for nutritional and potential therapeutic applications will depend heavily on emerging technologies that address current limitations in bioavailability, stability, and scalability. Two areas receiving particular attention are nano-delivery systems and biotechnological production platforms, which offer possible solutions for improving consistency and supporting sustainable supply.

Nano-delivery systems are being explored to mitigate the poor absorption and limited blood–brain barrier penetration of many seaweed compounds. Nanoparticles, liposomes, micelles, and biopolymer conjugates can encapsulate bio-actives such as fucoxanthin, phlorotannins, and fucoidan, protecting them from degradation in the gastrointestinal tract and enabling more controlled release. These systems may improve pharmacokinetic properties by enhancing solubility and prolonging circulation time, although evidence for targeted delivery to neural tissues remains limited. For example, fucoxanthin-loaded nanoparticles have shown improved antioxidant activity in preclinical models compared with free compounds [34]. Polysaccharide-based nanocarriers derived from alginate and carrageenan are also being investigated as both delivery vehicles and as bioactive agents, but their potential synergistic effects require further validation [39].

Biotechnological production provides a complementary strategy to ensure consistent and scalable supply of seaweed-derived compounds. Traditional harvesting is constrained by seasonal variability, geographic factors, and sustainability considerations. Advances in algal biotechnology—including controlled aquaculture, metabolic engineering, and synthetic biology—enable the production of extracts with more reproducible composition. Engineered microalgae and heterologous microbial systems are being developed to biosynthesize high-value compounds such as fucoxanthin, phlorotannins, and sulfated polysaccharides, reducing reliance on wild stocks and supporting environmental sustainability [28,35].

The integration of these technologies into marine-medicine and personalized-nutrition frameworks may support more standardized investigation of seaweed-derived bio-actives. Nano-delivery systems could help refine dosing strategies, while biotechnological production enhances supply chain resilience and traceability. Together, these approaches represent a shift in how marine resources are being explored for human-health applications, although their relevance for neurobiological outcomes will depend on rigorous evaluation in controlled human studies.

## 9. Conclusions

Seaweed-derived compounds exhibit diverse biological activities in preclinical systems; however, their clinical relevance remains unproven. Current evidence does not support their use as therapeutic agents for neurodegenerative diseases. Although these compounds show biological activity in vitro and in animal models, clinical neuroprotection has not been demonstrated, and their translational potential remains uncertain.

Their diverse chemical composition, including polysaccharides, phlorotannins, carotenoids, sterols, peptides, and Omega-3 fatty acids, supports multiple biological pathways implicated in oxidative stress, neuroinflammation, mitochondrial function, and protein homeostasis. These multi-targeted properties have motivated extensive preclinical investigation, but they do not establish therapeutic efficacy. Evidence from in vitro and in vivo studies highlights mechanistic interest rather than validated clinical benefit, and their long history of dietary use does not substitute for formal safety or efficacy evaluation.

Several challenges must be addressed before seaweed-derived bio-actives can be meaningfully assessed in clinical settings. Limitations in bioavailability, extract standardization, safety assessment, and regulatory classification remain major obstacles. Emerging technologies—such as nano-delivery systems, biotechnological production platforms, and personalized-nutrition approaches—may help address some of these issues, but their effectiveness must be confirmed through rigorous human studies.

Future research should prioritize standardized extraction and characterization protocols to ensure reproducibility, pharmacokinetic and toxicological profiling to establish safe dosage ranges, and well-designed clinical trials to evaluate efficacy in human populations. International collaboration will be essential to harmonize guidelines and support responsible investigation of these compounds.

Seaweed-derived compounds may eventually progress from traditional dietary components to candidates for targeted nutritional interventions, but any therapeutic implications will depend on robust clinical validation. Until such evidence is available, claims of neuroprotection or disease modification remain premature. Future research must therefore focus on pharmacokinetics, standardized extracts, and randomized clinical trials before therapeutic claims can be considered.

## Figures and Tables

**Table 1:** Major Seaweed-Derived Compounds and Their Neurological Activities.

Compound/Class	Seaweed Source	Species Name	Neurological Activity	Mechanism of Action	Model/Study Type	References
Fucoidan (polysaccharide)	Brown algae	*Fucus vesiculosus*, *Undaria pinnatifida*	Neuroprotection, anti-inflammatory	Inhibits microglial activation, reduces cytokines	In vitro, animal models	[3,4]
Phlorotannins (polyphenols)	Brown algae	*Ecklonia cava*, *Eisenia bicyclis*	Cognitive enhancement, antioxidant	Scavenges ROS, modulates cholinesterase activity	In vitro, rodent models	[5,6]
Fucoxanthin (carotenoid)	Brown algae	*Undaria pinnatifida*, *Sargassum horneri*	Anti-Alzheimer’s, neurogenesis	Reduces amyloid-beta aggregation, promotes neuronal survival	In vitro, zebrafish	[7,8]
Carrageenan (polysaccharide)	Red algae	*Chondrus crispus*, *Kappaphycus alvarezii*	Neuroprotective, anti-inflammatory	Modulates NF-κB pathway	In vitro	[9]
Omega-3 fatty acids	Green algae	*Ulva lactuca*, *Ulva prolifera*	Neurodevelopment, synaptic plasticity	Enhances membrane fluidity, neurotransmission	Human dietary studies	[10]

Note: All activities reported are from in vitro and/or animal studies.

**Table 2:** Summary of evidence for seaweed-derived bio-actives across experimental levels.

Compound/Class	Type of Evidence	Main Reported Outcomes	Major Limitations	Refs.
Fucoidan (brown algae)	In vitro; animal; limited human (indirect markers)	Antioxidant, anti-inflammatory, immunomodulatory effects; modulation of apoptosis and mitochondrial pathways	Poor oral bioavailability; high molecular weight limits absorption; uncertain CNS penetration; variability in sulfation patterns; species/seasonal variability; limited human data not related to neurodegeneration	[1,3,4,7,19,25,26]
Laminarin (brown algae)	In vitro; animal	Antioxidant activity; modulation of immune responses; effects on gut microbiota	Limited pharmacokinetic data; low stability; no CNS-related evidence; lack of standardized extracts	[27,28,29]
Ulvan (green algae)	In vitro; animal	Immunomodulation; antioxidant effects; gut–brain axis interactions	Structural heterogeneity; poor absorption; no human data; unclear dose–response; extraction variability	[15,27,29]
Phlorotannins (brown algae polyphenols)	In vitro; animal; limited human (surrogate endpoints)	Antioxidant activity; modulation of oxidative stress pathways; enzyme inhibition; anti-inflammatory effects	Low stability; rapid metabolism; limited bioavailability; uncertain CNS distribution; human outcomes indirect (fatigue, antioxidant status)	[5,6,11,21,30,31]
Fucoxanthin (carotenoid)	In vitro; animal; small pilot human studies	Antioxidant and metabolic effects; mitochondrial modulation; anti-inflammatory activity	Very low bioavailability; rapid metabolism; limited human data; no neurodegenerative endpoints; formulation-dependent absorption	[7,8,32,33,34]
Porphyran (red algae)	In vitro; animal	Antioxidant and immunomodulatory effects; gut microbiota modulation	No human studies; structural variability; limited pharmacokinetic data; uncertain CNS relevance	[27,29]
Carrageenan/Agar (red algae polysaccharides)	In vitro; animal; human (GI tolerance)	Gelling and prebiotic properties; immunomodulatory effects	GI discomfort in sensitive individuals; no CNS-related evidence; safety concerns at high doses; extraction variability	[9,27,29]
Alginate (brown algae polysaccharide)	In vitro; animal	Antioxidant and anti-inflammatory effects; use as delivery matrix	Not absorbed systemically; effects mostly indirect; no neurodegenerative endpoints; variability in composition	[27,28,29]
Sterols (e.g., fucosterol)	In vitro; animal	Antioxidant, anti-inflammatory, and membrane-modulating effects	Limited human data; uncertain bioavailability; potential oxidation; no CNS penetration data	[23,28]
Peptides (various species)	In vitro	Antioxidant and enzyme-modulating effects	Very limited evidence; no animal or human studies; rapid degradation; unclear physiological relevance	[24,35]
Omega-3 fatty acids from algae	In vitro; animal; human (general health)	Anti-inflammatory and metabolic effects; support for general brain health	Effects not specific to seaweed; neurodegenerative outcomes unproven; dose-dependent variability; oxidation sensitivity	[10,23,36]
Nano-delivery formulations	In vitro; animal	Improved solubility and stability; enhanced antioxidant activity; controlled release	Preclinical only; uncertain safety; no human data; unclear CNS targeting; formulation-dependent variability	[28,37]
Biotechnologically produced compounds	Preclinical; production studies	Standardized composition; scalable production; reduced environmental variability	Early-stage research; no clinical validation; regulatory uncertainty; strain-dependent differences	[20,38,39]

**Table 4:** Representative Seaweed Species, Major Extracts, Bioactivities, and Supporting References.

Seaweed Species	Major Extracts	Notes/Bioactivity	Refs.
*Fucus vesiculosus* (Phaeophyceae)	Fucoidan (sulfated polysaccharide), phlorotannins	Antioxidant, anti-inflammatory, anti-amyloid; widely studied for Alzheimer’s disease (AD) and Parkinson’s disease (PD)	[16,29]
*Undaria pinnatifida* (wakame, Phaeophyceae)	Fucoidan, fucoxanthin	Neuroprotective, anti-oxidative, supports autophagy and mitochondrial function	[16,32]
*Laminaria digitata* (Phaeophyceae, kelp)	Laminarin (β-glucan polysaccharide), alginate	Immunomodulatory, antioxidant, supports gut–brain axis	[29,68]
*Sargassum fusiforme* (Phaeophyceae)	Polysaccharides, fucoidan, sterols	Cognitive improvement in AD mouse models; cholesterol modulation	[61,69]
*Porphyra/Pyropia* spp. (nori, Rhodophyta)	Porphyran (sulfated polysaccharide), peptides	Antioxidant, anti-inflammatory, neuroprotective	[29,31]
*Ulva lactuca* (sea lettuce, Chlorophyta)	Ulvan (sulfated polysaccharide)	Immunomodulatory, antioxidant, potential neuroprotective effects	[15,29]
*Gracilaria* spp. (Rhodophyta)	Agar, carrageenan	Antioxidant, anti-inflammatory, gut microbiota modulation	[29,70]
*Ascophyllum nodosum* (Phaeophyceae)	Phlorotannins, fucoidan	Strong antioxidant and anti-inflammatory activity	[11,67]
*Chondrus crispus* (Irish moss Rhodophyta)	Carrageenan	Antioxidant, anti-inflammatory, stabilizing agent in formulations	[29,31]

## Data Availability

No new data were created or analyzed in this study. Data sharing is not applicable to this article.

## Abbreviations

The following abbreviations are used in this manuscript:

AD	Alzheimer’s Disease
Aβ	Amyloid Beta
AI	Artificial Intelligence
ALS	Amyotrophic Lateral Sclerosis
ADME	Absorption, Distribution, Metabolism, and Excretion
AMPA	α-Amino-3-Hydroxy-5-Methyl-4-Isoxazolepropionic Acid
ATP	Adenosine Triphosphate
BDNF	Brain-Derived Neurotrophic Factor
CaMKII	Calcium Influx, Activation of Kinases
CDK5	Cyclin Dependent Kinase 5
DHA	Docosahexaenoic Acid
DNA	Deoxyribonucleic Acid
DSHEA	Dietary Supplement Health and Education Act
EAE	Experimental Autoimmune Encephalomyelitis
EMA	European Medicines Agency
EPA	Eicosapentaenoic Acid
EU	European Union
FDA	Food and Drug Administration
GSK-3β	Glycogen Synthase Kinase 3β
H_2_S	Hydrogen Sulfide
IL-1β	Interleukin-1 Beta
IL-6	Interleukin-6
IL-10	Interleukin-10
IND	Investigational New Drug
LTD	Long-Term Depression
LTP	Long-Term Potentiation
mPTP	mitochondrial Permeability Transition Pore
MS	Multiple Sclerosis
NF-κB	Nuclear Factor Kappa-Light-Chain-Enhancer of Activated B Cells
NMDA	N-Methyl-D-Aspartate
Nrf2	Nuclear Factor Erythroid 2-Related Factor 2
PD	Parkinson’s Disease
PUFAs	Polyunsaturated Fatty Acids
ROS	Reactive Oxygen Species
SH-SY5Y	Human Neuroblastoma
TNF-α	Tumor Necrosis Factor-Alpha
